# Label-free liquid chromatography mass spectrometry analysis of changes in broiler liver proteins under transport stress

**DOI:** 10.1371/journal.pone.0311539

**Published:** 2024-10-28

**Authors:** Alessio Di Luca, Francesca Bennato, Andrea Ianni, Camillo Martino, Michael Henry, Paula Meleady, Giuseppe Martino

**Affiliations:** 1 Department of Bioscience and Technology for Food Agro-Food and Environmental Technology, University of Teramo, Teramo, Italy; 2 Department of Veterinary Medicine, University of Perugia, Perugia, Italy; 3 National Institute for Cellular Biotechnology, Dublin City University, Dublin, Ireland; 4 School of Biotechnology, Dublin City University, Dublin, Ireland; University of Hawai’i at Manoa, UNITED STATES OF AMERICA

## Abstract

Transportation duration and distance are significant concerns for animal welfare, particularly in the poultry industry. However, limited proteomic studies have investigated the impact of transport duration on poultry welfare. In this study, mass spectrometry based bottom up proteomics was employed to sensitively and impartially profile the liver tissue proteome of chickens, addressing the issue of animal stress and welfare in response to transportation before slaughter. The liver exudates obtained from Ross 508 chickens exposed to either short or long road transportation underwent quantitative label-free LC-MS proteomic profiling. This method identified a total of 1,368 proteins, among which 35 were found to be significantly different (p < 0.05) and capable of distinguishing between short and long road transportation conditions. Specifically, 23 proteins exhibited up-regulation in the non stressed group, while 12 proteins showed up-regulation in the stressed group. The proteins identified in this pilot study encompassed those linked to homeostasis and cellular energetic balance, including heat shock proteins and the 5′-nucleotidase domain-containing family. These results contribute to a deeper understanding of the proteome in broiler liver tissues, shedding light on poultry adaptability to transport stress. Furthermore, the identified proteins present potential as biomarkers, suggesting promising approaches to enhance poultry care and management within the industry.

## Introduction

Poultry experience a multitude of concurrent stressors during transportation, where the duration of transportation to slaughter emerges as a significant animal welfare concern [1]. Environmental factors such as temperature, vibration, noise, social disruption, water deprivation, fasting, and the length of the journey are the primary contributors affecting the intensity of this stress. The resulting negative effects encompass a range of issues, including diminished bird welfare, weight loss, immune response alterations, changes in behaviour, bird injuries, reduced meat yield, and quality, and in extreme cases, bird mortality [2–6]. These issues, in turn, lead to economic losses for the poultry industry due to the downgrading of carcasses [7]. All the stressors experienced during transportation can collectively impact poultry behaviour and physiology, a phenomenon well-documented in chickens [8–11]. For instance, elevated levels of corticosterone have been observed in broilers [12, 13], and transportation of broilers has been shown to alter their immune response [14, 15]. Additionally, stress can exacerbate endogenous microbial contamination, thereby increasing the risk to consumers of poultry meat [16]. Conversely, the effects of transportation on protein expression have received limited attention in the literature. Liu et al. [17] employed tandem mass tag (TMT) quantitative proteomic analysis to explore the impact of transport stress on liver iron metabolism in broilers. Their study revealed increased levels of iron ions in the liver following transport stress, along with notable changes in the expression levels of several proteins involved in iron metabolism.

The liver is crucial for maintaining the animal body’s homeostasis. It plays a pivotal role in producing circulatory proteins, conducting essential metabolic functions, synthesizing proteins, facilitating detoxification, and enabling substance exchange, as well as contributing to immunological and inflammatory responses [18, 19]. Due to these characteristics, the liver serves as an ideal matrix for studying adaptation mechanisms under transport stress.

Despite the potential of proteomics approaches, this technology has seen limited use in elucidating the molecular processes involved in the response to liver stress and in discovering biomarkers that could enhance animal production sectors [17, 20]. Proteomics allows for the comprehensive analysis of proteins in cells, tissues, or organs, offering a powerful method to assess the roles of proteins under various conditions (such as diseases, stress, breeds, etc.) in an unbiased manner, thereby providing an approach to identify potential biomarkers [21]. Label-free quantitative proteomics is a technique that demands a lower sample volume and can accurately estimate protein abundance over a wider dynamic range compared to labeling proteomics techniques [22, 23]. This technique has already been successfully applied to investigate the molecular processes related to stress adaptability following transport stress in pig muscle [24] and the temporal response to heat stress in broiler liver [20]. In this study, we employed a global proteomics approach to investigate the molecular processes underlying the liver’s response to the duration of transportation before slaughter. Our aim was to quantify variations in protein abundance between two time-of-transit conditions, namely short and long road transportation from the farm to the processing plant.

## Materials and methods

### Ethics statement

Animal care procedures for the experiment were carried out in compliance with a research protocol adhering to the Italian and European Community Directive [25]. The birds were slaughtered at a local poultry farm following standard procedures, and therefore, an additional ethical statement was deemed unnecessary.

### Animals and samples

Twelve broilers of the same genetic type (Ross 508) were included in this study. To evaluate the effect of transport on chickens during a typical 2.5 hours journey from the farm to the processing plant, six chickens underwent a long road transportation (approximately 2.5 hours, representing the stressed group), while the remaining six experienced a reduced stress situation with a short road transportation (about 10 minutes, representing the non-stressed group) from the farm to the processing plant on the day of slaughter. These broilers (Ross 508) were housed in an indoor pen within an environmentally controlled room on a farm situated in the Abruzzo region of Italy, as previously detailed in a prior publication [26]. The chickens were managed in accordance with EU legislation, and standard neon lighting was employed. All the broilers used in this study were in good health. The specific procedures applied for the study’s design have already been detailed in a prior publication [26]. The management of all chickens included in this study, including housing, feeding, and handling, was consistent, ensuring that the only distinction between the two groups was the duration of transportation. On the day of slaughter, the broilers were placed in chicken transport cages with dimensions measuring 95.5 cm x 57 cm x 27.5 cm. They were then divided into two groups. In the first group, to replicate a typical 2.5 hours journey from the farm to the processing plant, one cage was loaded onto an open truck and driven for approximately 2.5 hours (representing the stressed birds) before arriving at the processing plant. The second group followed the standard 10 minutes journey from the farm to the processing plant, representing a reduced stress situation (non-stressed birds). To reduce variables that could influence the stress of the birds, such as heat stress, and to focus on the effect of transportation, the transport simulation took place in January when outside temperatures ranged from 4 to 8°C in the morning of the transport day. No signs of mortality were observed among the chickens following the long road transportation from the farm to the processing plant. Both groups’ journeys were carefully synchronized to arrive at the processing plants at approximately the same time. At the conclusion of the journey, both the stressed and non-stressed bird groups were unloaded, electrically stunned, and subsequently slaughtered at the commercial processing plant of the company. This occurred after a 12 hours fasting period at the conclusion of their regular production cycle (commercial weight: 3.5 ± 0.3 kg, live weight). Following slaughter, liver samples were collected from six chickens in each group, totaling 12 chickens. These samples were collected post-evisceration, appropriately labeled, and stored at 4°C for subsequent protein extraction (exudate) on the same day as slaughter.

### Protein extraction, digestion and label-free LC-MS/MS analysis

We utilized six broilers that underwent a long road transportation lasting approximately 2.5 hours (representing stressed birds) and another six broilers that experienced a short road transportation of about 10 minutes (non-stressed birds) from the farm to the processing plant on the day of slaughtering. Protein extraction from the exudate was performed on the same day as slaughter, following the protocol outlined in Di Luca et al. [24]. The exudate collected from the chicken livers using this method ranged in volume from approximately 10 μl to 520 μl, with an average of about 137.7 μl. Protein concentration was determined in triplicate using the Bradford method and a BSA standard [27].

Protein purification and tryptic digestion were carried out following the Filter Aided Sample Preparation (FASP) protocol [28, 29]. Peptides from all samples were collected for Nano LC–MS/MS analysis. The analysis was conducted using Ultimate 3000 RSLCnano systems coupled in-line with an Orbitrap Fusion Tribrid^™^ mass spectrometer (Thermo Scientific, USA).

The MS raw files were merged and subjected to database searches for protein identification using Proteome Discoverer v.2.2 (Thermo Fisher Scientific, USA). LC-MS proteomics data analysis was performed using Progenesis QI for proteomics v.2.0 (NonLinear Dynamics, Newcastle upon Tyne, UK). The searches were carried out by identifying proteins in a UniProtKB database, which contained 32,426 protein sequence entries and was downloaded in August 2020. This database contained proteins from *Gallus gallus* (Chicken).

After alignment and normalization, a statistical analysis between groups was conducted using the software integrated into Progenesis QI for proteomics. The normalized peptide abundance across groups was compared using one-way ANOVA. Proteins were considered differentially expressed if they met the following criteria: 1) ANOVA values with a *p*-value of <0.05, 2) proteins with at least 2 matched peptides, and 3) a fold difference in abundance of ≥1.5.

The methods and parameters employed for MS analysis and protein identification are in accordance with the description provided by Di Luca et al. [30].

### Protein classification

The 35 proteins that exhibited significant differential expression were subjected to classification analysis using the PANTHER (Protein Analysis Through Evolutionary Relationships) database system, release 14.1 (http://www.pantherdb.org/) [31], with default parameters for the analysis. Data related to biological processes were collected.

The functional interpretation of differentially abundant proteins was conducted in Cytoscape (http://www.cytoscape.org/) [32] using the ClueGO plugin (http://www.ici.upmc.fr/cluego/) [33]. Gene enrichment analysis was performed on the Gene Ontology (GO)—Biological Process (BP) branch, with data from the November 2019 release. The analysis involved the following parameters: GO hierarchy level from 3 to 8, a minimum of 2 genes per GO term, a minimum of 2% input genes associated with the functional term, a GO term network connectivity (Kappa score) of 0.40, and a right-sided hypergeometric test. These adjustments were made to enhance the functional association process. The gene enrichment analysis and the organization of pathway term networks were based on Gallus gallus-specific functional annotations from May 2020, as previously described [30]. GO:BP terms with a Benjamini–Hochberg corrected *p-*value of <0.05 were considered statistically over-represented. Additionally, a separate pathway enrichment analysis was carried out using the ClueGO plugin with the KEGG pathway database from the November 2019 release.

In silico Protein-Protein Interaction (PPI) analysis of the differentially abundant proteins was performed using the STRING v.11.5 (Search Tool for the Retrieval of Interacting Genes/Proteins) database (https://string-db.org/) [34]. For this analysis, a *Gallus gallus* specific interactome with a STRING combined score greater than 0.7 was employed.

The parameters used for all analyses were consistent with those described in previous studies [30, 35].

## Results

### Quantification and statistical analysis of differentially expressed proteins in stressed and non-stressed chicken

Label-free LC-MS analysis was employed to quantify proteins obtained from the liver of chickens following centrifugation (exudate). The aim was to analyze protein changes between stressed and non-stressed birds (Ross 508). A total of 1,368 proteins were identified across all 12 samples, corresponding to the accurate identification of 7,264 peptides (S1 Table).

**Fig 1:**
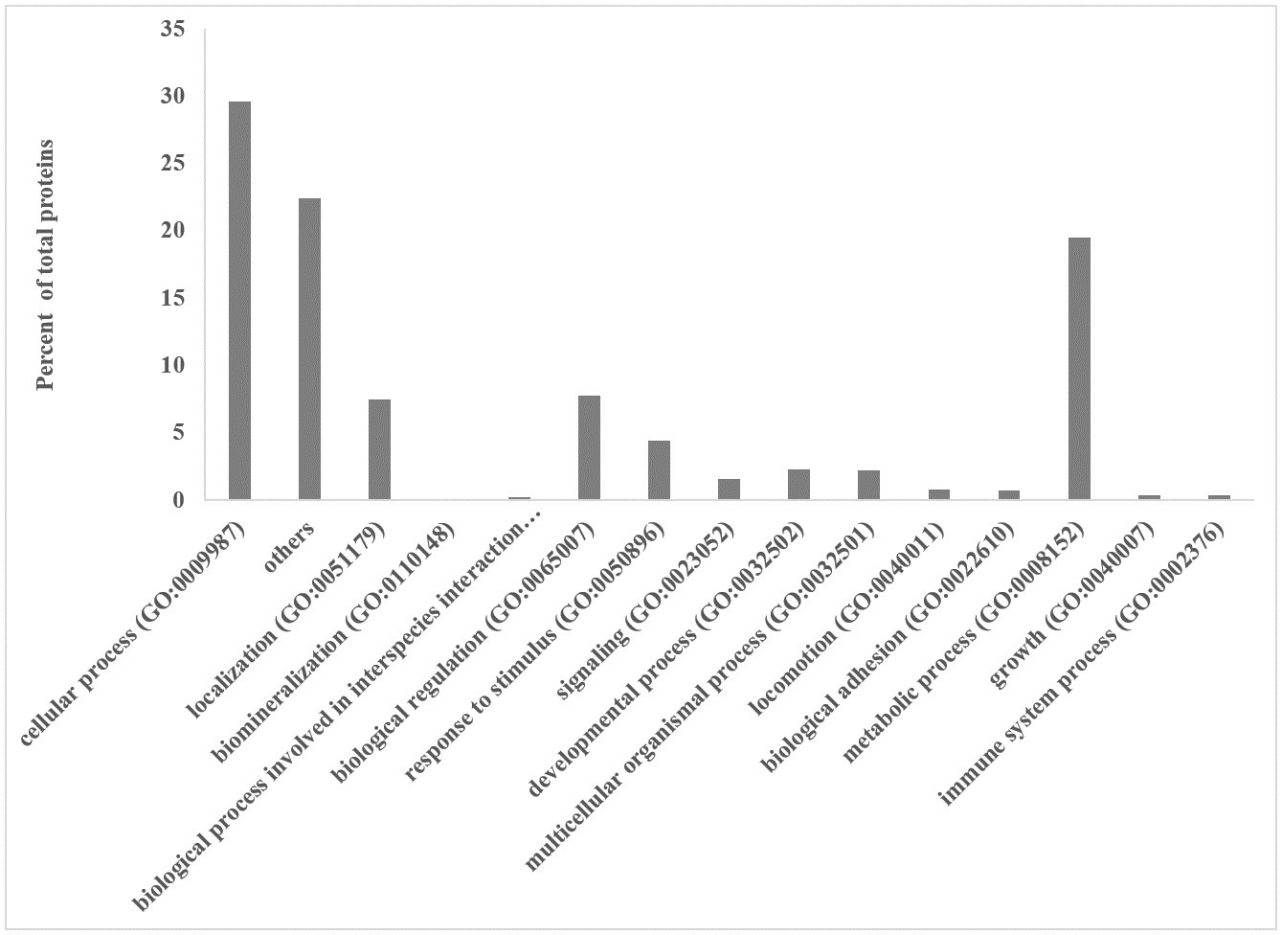
Proteins classification according to their biological processes (over 1,368 identified proteins; S1 Table).

The PANTHER (Protein ANalysis THrough Evolutionary Relationship) system (http://www.pantherdb.org) was utilized for gene ontology (GO) analysis of all 1,368 proteins. Gallus gallus genome annotations were used as the background for this analysis. Fig 1 shows the categorization of biological processes for all the identified proteins. These proteins were primarily involved in cellular processes (29.6%), metabolic processes (19.5%), biological regulation (7.8%), and localization (7.5%).

Progenesis QI for Proteomics was employed to explore the differences at the proteome level between the two groups of birds (stressed versus non-stressed). The thresholds used for significance were a *p*-value of ≤0.05, a fold change of ≥1.5, and proteins with at least ≥2 peptides matched. A total of 35 proteins (from 121 peptides) were identified as differentially expressed between the bird groups. Among these, 23 proteins (from 80 peptides; 66.12%) were up-regulated in the non-stressed group, while 12 proteins (from 41 peptides; 33.88%) were up-regulated in the stressed group, as illustrated in Table 1.

**Table 1:** Thirty-five proteins identified with altered levels of expression in response to transport stress (stressed) compared to non-stressed birds, as revealed by label-free MS/MS analysis using progenesis QI for proteomics.

UniProt^a^	Gene Name	Description	Peptides^b^	Score^c^	*p*-value	Fold change	Highest condition^d^
**a**							
A0A1D5NYK7	MSN	Moesin	9	30.55	0.00099	1.98	NON STRESSED
E1C2S1	TLN1	Talin-1	8	22.67	0.00005	1.60	NON STRESSED
R4GM10	ALDOC	Fructose-bisphosphate aldolase	7	17.77	0.00315	2.64	NON STRESSED
A0A1D5Q032	CAP1	Adenylyl cyclase-associated protein	5	10.89	0.00153	1.61	NON STRESSED
E1C2P9	KTN1	Kinectin	5	14.47	0.01331	1.65	NON STRESSED
Q90WF1	FLN	Filamin	5	14.16	0.02964	1.67	NON STRESSED
Q71SG2	CRMP2A	Collapsin response mediator protein-2A	4	12.70	0.00475	1.63	NON STRESSED
F1NT57	AKR1B1	Aldo_ket_red domain-containing protein	3	7.05	0.00101	1.72	NON STRESSED
A0A1D5PCT4	CORO1C	Coronin	3	5.89	0.00307	1.53	NON STRESSED
A0A1D5PDK5	IGSF9	Transgelin 2 OS = Gallus gallus	3	7.22	0.00521	1.83	NON STRESSED
Q5ZKI1	CLIC2	Chloride intracellular channel protein	3	7.72	0.01151	1.92	NON STRESSED
D2Z1L9	LASP1	LIM and SH3 protein 1	3	6.82	0.01587	1.64	NON STRESSED
E1BR38	ASS1	Argininosuccinate synthase	2	3.03	0.00208	2.15	NON STRESSED
O42134	CAPNS1	Calpain small subunit (Fragment)	2	3.54	0.00381	1.90	NON STRESSED
Q5ZI99	ARPC1B	Actin-related protein 2/3 complex subunit	2	6.14	0.00447	2.65	NON STRESSED
A0A1D5PPF8	HSPA4	Heat shock protein family A (Hsp70) member 4	2	5.26	0.00495	1.56	NON STRESSED
A0A1L1S0C5	SNX1	Sorting nexin 1	2	4.51	0.00737	1.76	NON STRESSED
A0A1D5NYG3	FLNB	Filamin B	2	7.54	0.00866	1.54	NON STRESSED
Q5F3C9	SH3BGRL	SH3 domain-binding glutamic acid-rich-like protein	2	5.40	0.00937	1.80	NON STRESSED
H9KYX6	SELENBP1	Methanethiol oxidase	2	4.49	0.01669	1.65	NON STRESSED
F1P2A6	ADA	Adenosine deaminase	2	4.97	0.02200	1.63	NON STRESSED
A1IMF0	UCH-L1	Ubiquitin carboxyl-terminal hydrolase	2	4.78	0.02672	2.52	NON STRESSED
E1C9F4	PSPH	Phosphoserine phosphatase	2	4.16	0.04071	2.47	NON STRESSED
**b**							
F1NMC3	MTHFD1	C-1-tetrahydrofolate synthase, cytoplasmic	6	19.06	0.00011	1.63	STRESSED
E1BXI0	BBOX1	Gamma-butyrobetaine hydroxylase 1	5	14.00	0.01299	2.16	STRESSED
F1NSB6	ANKFY1	Ankyrin repeat and FYVE domain containing 1	4	10.83	0.00791	2.98	STRESSED
E1C8W4	USP5	Ubiquitin carboxyl-terminal hydrolase	4	8.31	0.00957	2.51	STRESSED
F1NCR3	NT5C2	Cytosolic purine 5’-nucleotidase	4	9.12	0.01252	4.16	STRESSED
E1C1B4	GALK1	Galactokinase 1	4	13.62	0.01513	2.71	STRESSED
E1C771	FGGY	FGGY carbohydrate kinase domain containing	3	7.87	0.00069	5.76	STRESSED
A0A1D5PD80	NT5DC4	5’-nucleotidase domain containing 4	3	8.87	0.03482	2.94	STRESSED
A0A1D5PYP4	TTPA	Alpha tocopherol transfer protein	2	4.58	0.00559	4.69	STRESSED
A0A3Q2U869	PFKFB4	6PF2K domain-containing protein	2	5.85	0.01205	1.62	STRESSED
A0A452J7Z4	PRPS2	Ribose-phosphate pyrophosphokinase 2	2	6.80	0.01454	3.28	STRESSED
F1P5U4	NAT	Arylamine N-acetyltransferase	2	6.91	0.01550	2.62	STRESSED

23 proteins were up-regulated in the non-stressed chickens (Table 1a) while 12 proteins were up-regulated in stressed chickens (Table 1b). Key details for each protein include:

^a^) Accession number in the UniProt database.

^b^) Peptides used for quantitation.

^c^) SEQUEST score.

^d^) Indication of whether the proteins were up-regulated in non-stressed or stressed chickens."

### Functional annotation of significantly different proteins

**Fig 2:**
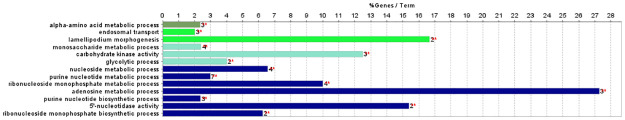
Gene enrichment analyses (Gene ontology-Biological process) of the proteins that are differentially expressed between the stressed and non-stressed groups. The percentage of input proteins found associated with respect to the number of proteins directly annotated with the functional term are represented by bars. The number of input proteins related to the term are next to each bar. Bars that share the same color are clustered in the same functional group (see Table 2 for details).

GO and KEGG pathway annotations were utilized to functionally annotate the differentially abundant proteins identified between bird groups (stressed versus non-stressed). Thirteen GO:BP terms corresponding to 14 differentially abundant proteins were retrieved (Table 2, Fig 2). The most prevalent biological processes included adenosine metabolic process (27.27%), lamellipodium morphogenesis (16.67%), 5’-nucleotidase activity (15.38%), carbohydrate kinase activity (12.5%), and ribonucleoside monophosphate metabolic process (10%).

**Table 2:** Over-represented biological processes (GO:BP) associated with up- or down-regulated proteins in response to transport stress.

GOID	Description	Functional group^1^	*p*-value^2^	% of associated proteins^3^	N. of proteins	Up or down regulated proteins ^4^
GO:1901605	alpha-amino acid metabolic process	Group0	0.00445	2.34	3	[ASS1↑, MTHFD1↓, PSPH↑]
GO:0016197	endosomal transport	Group1	0.00602	2.01	3	[ANKFY1↓, CORO1C↑, SNX1↑]
GO:0072673	lamellipodium morphogenesis	Group1	0.00602	16.67	2	[CORO1C↑, SNX1↑]
GO:0005996	monosaccharide metabolic process	Group2	0.00212	2.40	4	[ALDOC↑, FGGY↓, GALK1↓, PFKFB4↓]
GO:0019200	carbohydrate kinase activity	Group2	0.00212	12.50	3	[FGGY↓, GALK1↓, PFKFB4↓]
GO:0006096	glycolytic process	Group2	0.00212	4.00	2	[ALDOC↑, GALK1↓]
GO:0009116	nucleoside metabolic process	Group3	0.00001	6.56	4	[ADA↑, NT5C2↓, NT5DC4↓, PRPS2↓]
GO:0006163	purine nucleotide metabolic process	Group3	0.00001	2.98	7	[ADA↑, ALDOC↑, GALK1↓, MTHFD1↓, NT5C2↓, NT5DC4↓, PRPS2↓]
GO:0009161	ribonucleoside monophosphate metabolic process	Group3	0.00001	10.00	4	[ADA↑, NT5C2↓, NT5DC4↓, PRPS2↓]
GO:0046085	adenosine metabolic process	Group3	0.00001	27.27	3	[ADA↑, NT5C2↓, NT5DC4↓]
GO:0006164	purine nucleotide biosynthetic process	Group3	0.00001	2.36	3	[ADA↑, MTHFD1↓, PRPS2↓]
GO:0008253	5’-nucleotidase activity	Group3	0.00001	15.38	2	[NT5C2↓, NT5DC4↓]
GO:0009156	ribonucleoside monophosphate biosynthetic process	Group3	0.00001	6.25	2	[ADA↑, PRPS2↓]

^1^Groups of closely related terms

^2^Benjamini-Hochberg corrected *p*-values

^3^Percentage of input proteins found associated with respect to the number of proteins directly annotated with the functional term

^4^↑: protein up-regulated in non-stressed chickens compared to stressed chickens; ↓: protein up-regulated in stressed chickens compared to non-stressed chickens.

**Fig 3:**
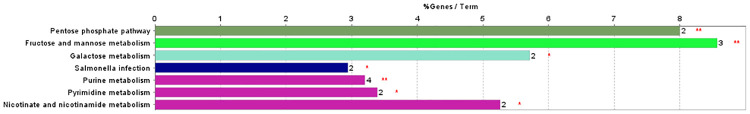
Gene enrichment analyses (KEGG pathway database) of the proteins that are differentially expressed between the stressed and non-stressed groups. The percentage of input proteins found associated with respect to the number of proteins directly annotated with the functional term are represented by bars together with the number of input proteins related to the term (see Table 3 for details).

Separate KEGG pathway analysis of all the differentially identified proteins in chicken liver exudate was performed and is presented in Table 3 and Fig 3. Seven KEGG pathways were identified, involving 10 proteins. These proteins were primarily associated with fructose and mannose metabolism (8.57%), pentose phosphate pathway (8%), galactose metabolism, nicotinate (5.71%), and nicotinamide metabolism (5.26%).

**Table 3:** Over-represented KEGG pathways associated with up- or down-regulated proteins in response to transport stress.

GOID	Description	Functional group^1^	*p*-value^2^	% of associated proteins^3^	N. of proteins	Up or down regulated proteins ^4^
KEGG:00030	Pentose phosphate pathway	G0	0.007	8.00	2	[ALDOC↑, PRPS2↓]
KEGG:00051	Fructose and mannose metabolism	G1	0.001	8.57	3	[AKR1B1↑, ALDOC↑, PFKFB4↓]
KEGG:00052	Galactose metabolism	G2	0.010	5.71	2	[AKR1B1↑, GALK1↓]
KEGG:05132	Salmonella infection	G3	0.028	2.94	2	[ARPC1B↓, FLNB↑]
KEGG:00230	Purine metabolism	G4	0.010	3.20	4	[ADA↑, NT5C2↓, NT5DC4↓, PRPS2↓]
KEGG:00240	Pyrimidine metabolism	G4	0.010	3.39	2	[NT5C2↓, NT5DC4↓]
KEGG:00760	Nicotinate and nicotinamide metabolism	G4	0.010	5.26	2	[NT5C2↓, NT5DC4↓]

^1^Groups of closely related terms

^2^Benjamini-Hochberg corrected *p*-values

^3^Percentage of input proteins found associated with respect to the number of proteins directly annotated with the functional term

^4^↑: protein up-regulated in non-stressed chickens compared to stressed chickens; ↓: protein up-regulated in stressed chickens compared to non-stressed chickens.

### Protein protein interaction (PPI) analysis

**Fig 4:**
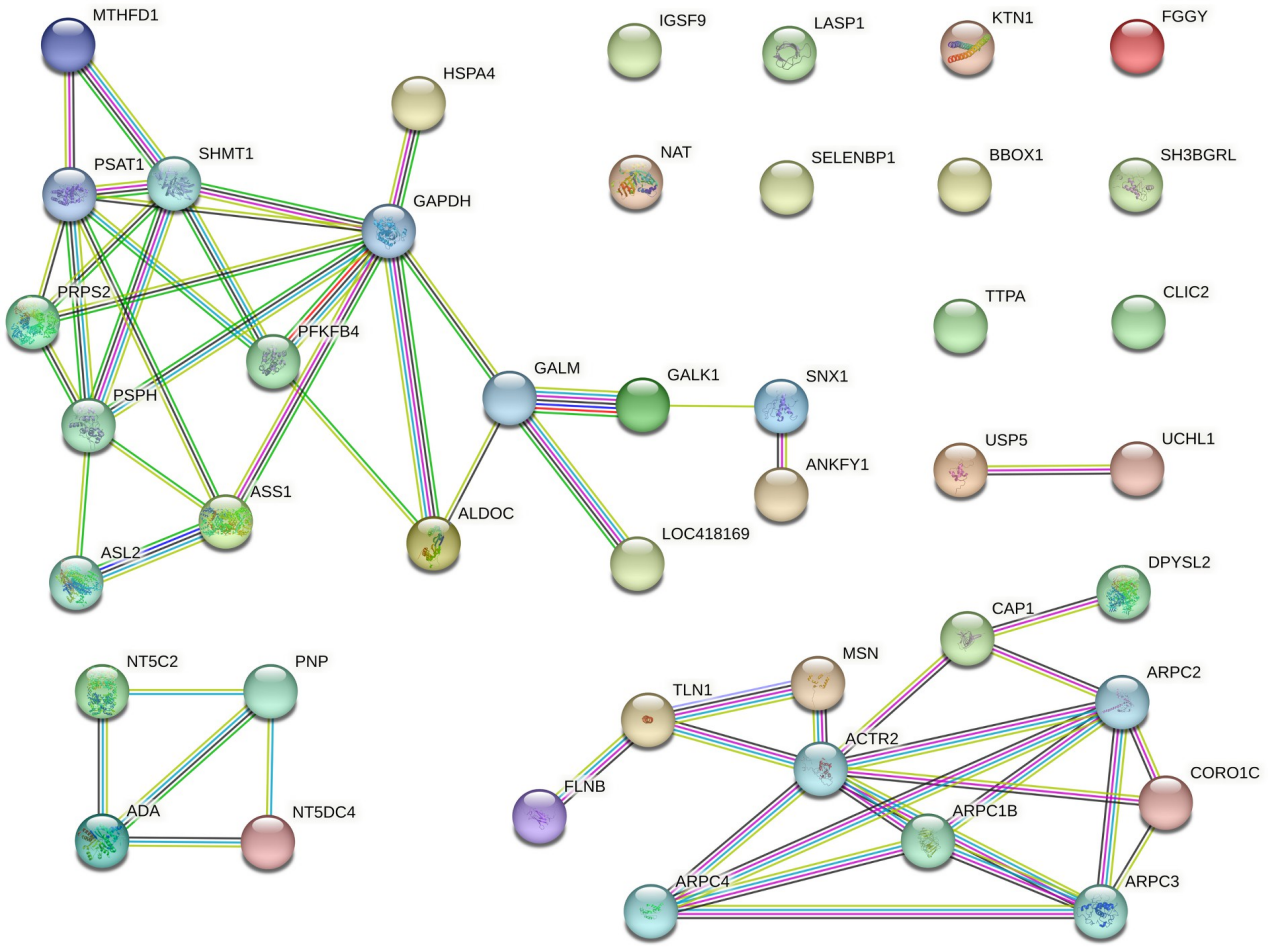
PPI network of the differentially expressed proteins (35 proteins) in chickens’ liver exudate. Proteins are represented by nodes. Association line colors: 1) magenta is experimentally determined; 2) dark green is gene neighborhood; 3) cyan is from curated databases, 4) black is coexpression; 5) purple is protein homology; 6) light green is textmining; 7) blue is gene co-occurrence.

STRING was used to reveal functional connections among the differentially expressed proteins (35 proteins). The analysis uncovered a connected protein network (Fig 4) consisting of: 1) one large module with 16 nodes (37.21%), 2) one module with 11 nodes (25.58%), 3) one small module with four nodes (9.3%), 4) one small component with two proteins (4.65%), and 10 singletons (23.26%), totaling 43 nodes. The PPI enrichment resulted in a *p*-value of < 3.66e-9 (22 expected edges vs. 55 detected edges), indicating that proteins are at least partially biologically connected. Notably, the majority of proteins in this network interacted with two or three other partners, with an average node degree of 2.56.

## Discussion

Transportation is indeed considered one of the most stressful challenges for birds [36]. While there are conflicting reports on the impact of transport time on broiler welfare and meat quality parameters, consensus exists regarding the association of high mortalities with prolonged transportation [7, 37]. In chickens, blood metabolites such as corticosterone and leukocyte counts serve as sensitive indicators of stress in response to transport time [11]. Corticosterone, released in response to stressors through the activation cascade of the hypothalamic pituitary adrenal axis, can indicate the birds’ stress level at the time of blood collection [38]. However, factors like handling, fear of humans, and the stress of capture can compromise the meaningfulness of stress assessments, potentially elevating corticosterone levels [39, 40]. Non-invasive measurements of corticosterone in excreta and feathers have been explored, but these methods come with limitations. Challenges include difficulty in collecting samples from individual birds, sample dilutions, and variations in feather sample types [41–43]. The development of objective criteria for evaluating chicken stress remains an area in need of improvement. While there is limited research on the effects of broiler transportation on tissue protein expression, Hazard et al. [44], using two-dimensional gel electrophoresis, identified 29 proteins primarily involved in cytoskeletal structure and carbohydrate metabolism. Similarly, Zanetti et al. [45], employing a similar approach, identified two proteins in chicken breast muscle that significantly changed in response to the duration of transit before slaughtering. When cells respond to stimuli, such as transport stress, they can undergo perturbations that lead to alterations in protein content within tissues like muscle or liver. These changes reflect the biochemical processes employed by the organism to cope with stressors [46]. Therefore, gaining a deeper understanding of the biological processes triggered by transport stress in chickens could yield biomarkers and valuable insights to mitigate the adverse effects of stressors. This achievement would be particularly significant for the poultry industry as a whole. To pursue this objective, we capitalized on advancements in proteomics technologies, comparing the proteome of broilers in response to the duration of transit before slaughtering. We used *post mortem* exudate collected from liver tissue following centrifugation as our substrate for analysis.

The identification of a substantial number of proteins in the liver of chickens enhances our comprehension of their response to transport stress. In our study, a total of 1,368 different proteins were identified. A comparable study on pig muscle exudate also identified a similar number of proteins (1,464 proteins), with the majority being involved in similar biological processes such as cellular and metabolic processes, biological regulation, and response to stimuli [24]. Our findings indicate a significant impact of transport stress on the chicken proteome, with 35 proteins (12 up-regulated and 23 down-regulated in birds stressed following long road transportation) implicated in the response to transport stress. Animal transport stress can disrupt cellular protein expression and composition, compromising proper regulation of protein homeostasis (proteostasis) [47]. Impaired proteostasis, linked to various diseases, may result in the accumulation of protein aggregates in different cellular compartments. Cells have evolved various strategies to address the loss of proteostasis, including disaggregation of protein aggregates, refolding of misfolded proteins, and degradation when proper refolding is not feasible [48–50].

Heat shock proteins (HSPs) form a family crucial for preventing the formation of non-functional proteins and aiding in protein folding. They shield proteins from stressors like pH and temperature variations, often serving as indicators to monitor proteome stress [47, 51]. HSP70, the most prevalent member of the HSP family, was up-regulated in birds subjected to short road transportation (non-stressed) in our study. A similar protein pattern was observed in other studies on transport stress, whether in the *longissimus dorsi* of pigs [52, 53] or the pig heart [54]. In contrast, our previous study on transport stress in pigs revealed an up-regulation of HSPs in the diaphragm muscle of pigs undergoing long road transportation. The weaker activity of HSPs after transport stress was postulated to be balanced by increased degradation by the proteasome of unfolded or damaged proteins as a self-protection mechanism within the cells [24]. Under normal conditions, HSP70 is located in the cytoplasm, but following stress events, it translocates to the nucleus [55]. The down-regulation of HSP70 observed in our liver exudate (collected after centrifugation) suggests that HSP70 might be more active in the nucleus, a component that is difficult to extract through centrifugation, in response to transport stress. Indeed, it has been postulated that the HSP70 stress response in the liver is activated promptly after stress as an essential early trigger for tissue regeneration [56]. Intracellular HSP proteins play essential housekeeping roles under normal conditions, existing abundantly without the need for a priming step [57]. When cells face various sources of stress, a common pathway that activates Hsp70 involves ATP depletion. This depletion causes the denaturation of folded intracellular proteins, the formation of disordered protein aggregates, and compromised cell viability. The inducible chaperone activity of Hsp70 helps cells manage the increased load of misfolded proteins through a bind-and-release mechanism that facilitates refolding. This process depends on the intrinsic adenosine triphosphatase (ATPase) activity located at the N-terminus of Hsp70, which is catalyzed by other heat shock-responsive components, including members of the Hsp40 family and nucleotide exchange factors. The interactions with various Hsp40 proteins and nucleotide exchange factors enhance Hsp70’s substrate specificity [58, 59]. Additionally, Hsp70 can be released from stressed cells, functioning as an extracellular protein that serves as a paracrine signal [60].

Several proteins involved in carbohydrate metabolism were identified. For instance, proteins associated with the pentose phosphate pathway showed changes in abundance following transport stress in our study. The enzymes of the pentose phosphate pathway are essential for maintaining cytoplasmic NADPH concentrations, which provide the redox power necessary for antioxidant systems [61]. This suggests that alterations in carbohydrate metabolism could be crucial for cellular protection against reactive oxygen species (ROS) [62]. Furthermore, it indicates that cells may redirect carbohydrate flux from glycolysis to the pentose phosphate pathway to enable a rapid cellular response under stress conditions as in our study [63].

Proteins involved in the purine and pyrimidine metabolism pathways, such as cytosolic purine 5’-nucleotidase (NT5C2) and 5’-nucleotidase domain-containing 4 (NT5DC4), were up-regulated in the stressed chickens. Purine and pyrimidine nucleotides play crucial roles in biological processes, including the synthesis of RNA and DNA, as well as serving as energy carriers [64]. Maintaining balanced purine metabolism is crucial for mammalian cells to manage oxidative stress, as any dysregulation is linked to various disorders and diseases [65]. For instance, a deficiency in hypoxanthine-guanine phosphoribosyltransferase leads to hyperuricemia (high uric acid levels in the blood), which can cause clinical issues such as gout and renal failure [66]. Additionally, mutations or deficiencies in adenosine deaminase, an essential enzyme for purine degradation or salvage, increase susceptibility to infections and autoimmunity [67]. The 5′-Nucleotidase domain-containing (NT5DC) family, including members NT5C2 and NT5DC4, represents a well-conserved group of enzymes capable of catalyzing the hydrolysis of nucleotides within cells [68]. These proteins play a crucial role in metabolic regulation, cell replication, and maintaining cellular energy balance. They have been associated with severe pathologies such as human colon carcinoma, where NT5C2 activity is observed to increase compared to the activity measured in the apparently normal peritumoral tissue from the same patients [69, 70]. Moreover, the hyperactivity of these proteins has also been observed in the red cells of patients with Lesch–Nyhan syndrome [71]. These are widespread proteins whose presence has been detected in mammals (e.g., human, rat, pig), frog, invertebrate, fish, reptiles (e.g., turtle, snakes), and birds (e.g., chicken and goose) [72]. Birds’ liver exhibits high expression of these proteins, a phenomenon further heightened following a protein-rich diet or during liver regeneration [73]. The up-regulation observed in our study may suggest an increased demand for energy by the birds’ liver to cope with transport stress. Speculatively, these proteins may hydrolyze excess inosine monophosphate, crucial for adenosine triphosphate (ATP) synthesis, particularly when the cell energy charge is high [70, 74]. In addition, the liver is likely a crucial organ for supplying preformed purines to cells in other parts of the body [75]. Disturbances in pyrimidine and purine metabolites have been associated with liver fibrosis and cirrhosis [76]. The modulating role of gut microbiota in the metabolism of pyrimidine and purine has been observed in mice [77, 78]. Moreover, gut microbiota-derived purine metabolites, including eATP and xanthine, have been linked to the development of inflammatory bowel disease [79]. In our study, birds responding to transport stress may have experienced an inflammatory process involving the release of neuropeptides or other inflammatory mediators. Indeed, the central nervous system has the capacity to both produce and modulate general inflammatory reactions in response to stress [80], potentially influencing the metabolism of pyrimidine and purine.

Road transport is a multifactorial issue where a combination of stressors such as bird handling, loading, unloading, bird density, environmental conditions, transit duration, and feed withdrawal affects the animals’ well-being and the post-transport meat quality [7]. Due to the various components influencing the responses of birds to transportation, it is difficult to isolate the effect of transit duration from all other factors. To address this challenge, increasing the number of birds studied is necessary, although this is not always feasible due to methodological limitations, such as those in proteomics studies. The breed of the birds is another important factor influencing welfare during transportation and the final quality of the meat. For instance, local breeds often tolerate harsh environmental conditions, including transportation and pathogens, better than commercial hybrids [81, 82]. Differences in adaptation to transport have also been observed when comparing the effects between slow-growing and fast-growing chickens. Slow-growing chickens exhibit a lower capability to tolerate stress, which results in a reduction in meat quality [83]. The utilization of a larger dataset, including other important factors influencing the capability of the birds to tolerate stress, is pivotal in addressing this challenge.

In this study, several proteins have been identified as potential biomarkers to enhance poultry care and management within the industry. A common issue with new protein biomarkers is the high false discovery rate [84–86]. To address this, candidate biomarkers must be validated in large sample sets. Traditional immunoassay techniques, such as ELISA, have been employed for this purpose. However, these methods have some drawbacks: they are expensive, time-consuming, and specific antibodies for the proteins of interest are not always readily available. This limitation reduces the number of candidate biomarkers that can be tested. Recently, advancements in mass spectrometry (MS) analysis have led to the use of targeted quantitative proteomics for the validation of candidate protein biomarkers. One such technique, Selected Reaction Monitoring (SRM), is particularly effective for this purpose. SRM is a mass spectrometry method used for the targeted quantification of proteins and is known for its high sensitivity, speed, and absolute structural specificity of the analyte detected. These attributes make SRM and similar techniques especially suitable for high-throughput validation studies, including biomarker validation [87–89]. These advanced MS-based approaches can be utilized in future studies to validate the proteins of interest in larger sample sets and/or in different poultry breeds, thereby enhancing the reliability and applicability of these biomarkers in the poultry industry.

Despite this work being considered a pilot study and recognizing that proteomic differences should be confirmed and validated with other approaches in a larger number of samples, the identification of thirty-five differentially expressed proteins linked to transport stress in chickens suggests that this stressor likely impacted protein homeostasis and cellular energetic balance. These findings not only contribute to a deeper understanding of the biological processes affected by transport stress but also open avenues for potential biomarkers. The identified proteins could serve as markers to better assess the stress resistance of chickens post-transport, offering valuable support to poultry producers.

## Supporting information

S1 Table Full list of the 1,368 proteins identified by mass spectrometry in chicken liver exudate in the stressed and non-stressed chickens determined in Proteome Discoverer using SEQUEST HT algorithm. (XLSX)
